# Construction of Roller Compacted Concrete Dams in Hot Arid Regions

**DOI:** 10.3390/ma12193064

**Published:** 2019-09-20

**Authors:** Khaled H. Bayagoob, S. O. Bamaga

**Affiliations:** Department of Civil Engineering, College of Engineering, University of Bisha, Bisha 61922, Saudi Arabia; sbamaga@ub.edu.sa

**Keywords:** RCC dams, thermal analysis, placement schedule, placing temperature

## Abstract

Roller compacted concrete (RCC) dams are attractive to many water and energy corporations around the world due to their ease in construction and low construction cost. The hydration of cement and the climatic changes on the convective boundaries are the two main heat sources for the temperature rise in the roller compacted concrete dams. Thus, changing the RCC placement schedule according to climate conditions might eliminate the problem of thermal cracks. In this research, the RCC dam method was applied in an arid region; the Bisha state in Saudi Arabia was chosen as a case study. We found that RCC dam technology can be applied safely with an alternative solution, like selecting a suitable placement schedule and reducing the placing temperature of facing at upstream and downstream sides to overcome the risk of thermal cracks.

## 1. Introduction

At the beginning of the construction of roller compacted concrete (RCC) dams, the problem of thermal cracks was thought to have been eliminated due to the lower quantity of cement in the RCC mix compared to conventional concrete, which greatly reduces the thermal strains. Later on, it was discovered that attention must be paid to the tensile stresses in RCC dams, where the use of cooling pipes during construction of these dams is not possible as usually done in conventional concrete dams. In addition to other effects like the speed of construction, different RCC placing temperatures, and climatic conditions, may raise the temperature of the dam body. This dam body heat takes a long time to dissipate, causing tensile stresses. When these tensile stresses exceed the RCC tensile strength, cracks develop in the dam body. Thus, the design and construction of RCC dams necessitates accurate thermal analysis, considering the parameters that influence crack development most, such as RCC properties, ambient conditions, placing schedule, and placing temperature of RCC, especially in arid regions.

Many mathematical models have been proposed to predict the heat evolution and the resulting induced stresses in the roller compacted concrete gravity dams, taking into account the hydration heat and the environmental conditions. Saetta et al. [1], presented a finite element procedure for the stress-strain analysis in concrete structures exposed to time-variable environmental conditions. The developed numerical model was applied to the analysis of a gravity RCC dam taking into account the heat generation phenomenon, seasonal, and daily variations of temperature. A constant temperature of 6 °C was assumed for the upstream face when it comes in contact with storage water in the case of the thermal analysis. Carvera et al. [2,3] presented a numerical procedure for the simulation of the construction processes of the Urugu-1 RCC gravity dam. The behavior of concrete at an early age such as hydration, aging, creep, and damage were taken into account. The effect of the reservoir water temperature on the thermal response of the dam body was ignored. Luna and Wu [4] simulated the construction process of an RCC gravity dam in China using a developed 3D finite element program. The results showed that, the gravity loads overcome the induced thermal tension stress. Waleed et al. [5] studied the effect of the placement schedule on the thermal and structural response of RCC gravity dams. They concluded that the RCC placement schedule can optimize the location of maximum temperature and, finally, leading to decreasing the tensile stresses at the critical zone. Noorzaei et al. [6] and Jaffar et al. [7] developed and verified a finite element code on the Kinta RCC gravity dam in Malaysia. The study showed that, the crack index gives a good indication of cracking which was predicted in the downstream side of the dam. 

Currently, RCC dam technology is receiving more attention. Gaspar et al. [8] carried out a sensitivity analysis of some RCC properties on the thermal behavior of an RCC gravity dam. The study concluded that, the convection coefficient has negligible sensitivity indices with an existing relationship to the thermal conductivity coefficient. Huaizhi et al. [9] studied the time-varying characteristics of RCC arch dam structure and material properties due to its long lifespan considering the effect of the hydrostatic and thermal loads. Xiao-Fei et al. [10] developed a three-dimensional (3D) finite element relocating mesh model to simulate the construction process and the temperature field of RCC dams. The developed method reduced the number of finite elements and the nodes and hence saved the memory and run time with small errors. Li et al. [11] constructed a finite element model to analyze the stress and seepage fields of RCC dams. The study showed that the maximum tensile and compressive stresses occurs near the dam heel and toe, respectively. Mirzabozorg et al. [12] studied the effect of solar radiation on the thermo-structural response of an arch dam and concluded that the solar radiation increases the dam body tensile stresses. Cavusliet et al. [13] studied the effects of reservoir lengths on the earthquake response of an RCC dam. The study concluded that, the principal stresses and the horizontal displacements were increased if the reservoir length is extended. Zhang et al. [14] studied the effect of the heat of hydration on rock-fill concrete, the study indicated that, without any temperature control, construction efficiency was improved where the occurrence of cracking was prevented and, as such, the construction cost was reduced. Kuzmanovic et al. [15] developed a 3D numerical model to evaluate thermal stress in RCC dams. The study indicated that, cracks are usually developed close to the upstream or the downstream face of the dam. Žvanut et al. [16] presented thermal analysis results of a large concrete arch gravity dam taking into account the concrete, water, and the surrounding temperatures. 

It is clearly understood from the above studies that, the climate conditions and the evolved hydration heat have great effects on induced stresses in the bodies of RCC dams. Thus, in this research work, the construction suitability of RCC dams in arid regions in Saudi Arabia was studied taking into account the most influential parameters. Saudi Arabia weather is recognized as being very hot and dry in summer and mild dry in winter. Al-Khanaq dam in Bisha state, Aseer region, Saudi Arabia is one of four gravity dams that are planned for construction in the near future. These dams range from 12 to 34 m in height. Al-Khanaq dam is the largest among these dams and for this reason it was selected as the case study in this research. The actual daily recorded environmental temperatures obtained from related authorities were used in the analysis.

## 2. Al- Khanaq RCC Dam Construction

**Figure 1:**
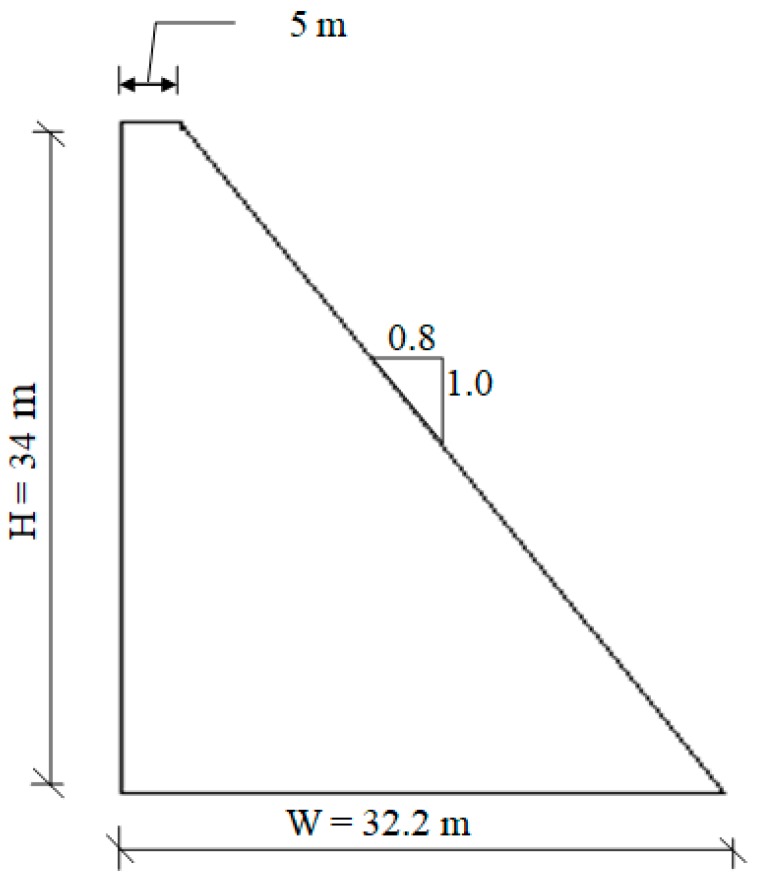
Al- Khanaq roller compacted concrete (RCC) dam cross-section.

**Figure 2:**
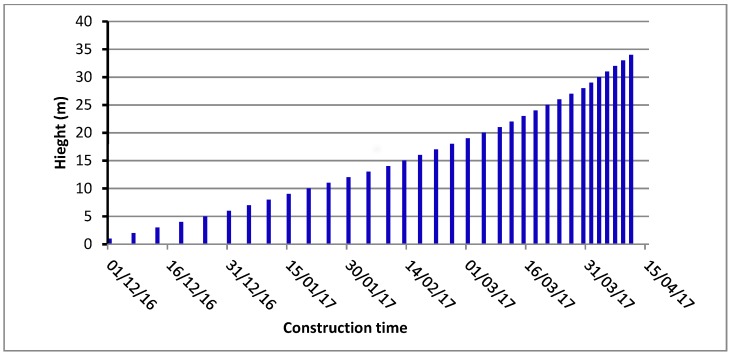
Construction progress of the highest block of Al-Khanaq dam.

Al-Khanaq dam is a gravity dam that to be constructed in Bisha state in Saudi Arabia. The maximum height of the dam is 34 m, and the crest length is 400 m. Based on the proposed dam cross-section shown in Figure 1, the total volume of the RCC material was estimated as 236,000 ton/m^3^. With an assumed RCC placement rate of 2000 m^3^/day, the dam construction time was estimated at 133 days. The construction of the dam was proposed to start on December 1, 2016 and finish on April 12, 2017. Figure 2 shows the suggested dam construction progress with time according to the RCC placement rate. The finite element modeling is developed based on the construction schedule shown in Figure 2.

## 3. Calculation Basis 

### 3.1. Mathematical Modeling

The governing partial differential equation for the heat transfer in a two-dimensional solid media is expressed in a general form as [17]

(1) $$k_{x}\frac{\partial^{2}T}{\partial x^{2}}+k_{y}\frac{\partial^{2}T}{\partial y^{2}}+\dot{Q}=\rho \;c\frac{\partial T}{\partial t}$$

where *k_x_* and *k_y_* are the coefficients of thermal conductivity in the *x* and *y* directions, respectively; $\dot{Q}$ is the rate of the introduced heat per unit volume; *c* is material’s specific heat; ρ is the material’s density; and *t* is the time.

There are two main boundary conditions at the external surfaces, which are expressed as

(2) $$\begin{array}{l} T=T_{p}\\ k_{x}\frac{\partial T}{\partial x}l_{x}+k_{y}\frac{\partial T}{\partial y}l_{y}+h\left(T_{s}-T_{f}\right) \end{array}$$

where *T_p_* is the known temperatures values on some boundaries; *l_x_* and *l_y_* are the direction cosines of the outward normal to the surface in the *x* and *y* directions, respectively; *h* is the film coefficient; *T_f_* is the ambient temperature; and *T_s_* is the unknown boundary temperatures [18].

### 3.2. Numerical Modeling

In this research, the used numerical solution scheme is based on the Taylor-Galerkin approach. Upon applying this approach, a system of differential equation was obtained [18]:

(3) $${\left[C\right]}^{e}\left\{\frac{\partial T}{\partial t}\right\}+{\left[K_{t}\right]}^{e}\;\left\{T\right\}-{\left\{F_{t}^{}\right\}}^{e}=0$$

where ${\left[C\right]}^{e}$ is the capacitance matrix, ${\left[K_{t}\right]}^{e}$ is the heat matrix, and ${\left\{F_{t}\right\}}^{e}$ is the total load heat vector. 

The general form of the solution in Equation (3), using the finite difference approximation with the backward difference method in the time domain, is given by [18]

(4) $$\left(\frac{1}{\Delta t}[C]-[K_{t}]\right)\{\Delta T\}={\left\{F_{t}\right\}}_{b}$$

where {Δ*T*} represents the temperature changes at the nodal points with respect to time change Δ*t*, which depends mainly on the value of the heat load vector ${\left\{F_{t}\right\}}_{b}$. This temperature change vector is used to evaluate the temperatures at the new time stage b:

(5) $${\left\{T\right\}}_{\left(b\right)}={\left\{T\right\}}_{\left(a\right)}+\left\{\Delta T\right\}$$

where ${\left\{T\right\}}_{a}$ and ${\left\{T\right\}}_{b}$ are {T} at time a and b, respectively.

The hydration heat of the RCC material is the main heat source in the dam’s body, which is represented by $\dot{Q}$ in Equation (1). The adiabatic model is widely used in the simulation of the heat of hydration in massive concrete structures. 

The rate of the heat of hydration $\dot{Q}$ in Equation (1) is given by [19]:

(6) $$\dot{Q}=c\rho T_{\max}^{}\alpha \;e^{-\alpha t}$$

where *T_max_* is the maximum temperature increase of concrete, α is a parameter that represents the heat generation rate, and *t* is the time in hours. In this research, Equation (6) was used for the calculation of the rate of heat of hydration, where *T_max_* and *α* are taken as 18.0 °C and 0.025, respectively, as the average values for RCC reported in literature [6].

### 3.3. Stress Analysis

After the thermal analysis of every RCC stage (lift) of construction, linear elastic was used, considering the variation in the RCC elastic modulus *E_c_* with time, which is expressed as [20]

(7) $$E_{c}(t)=k{\left(\infty \right)}_{}\;E_{c}\;epx\;(at^{b})$$

where *E_c_*(*t*) time dependent modulus in GPa, *E_c_* final modulus of elasticity in GPa, *t* concrete age in days, a and b are model parameters, *k(∞)* is the multiplier at infinite age set to 1.2, *E_c_* is the final elastic modulus, and *a* and *b* are the model parameters, which are set to –4.75 and –0.55, respectively.

The compressive strength variation with time is calculated using the relation of the CEB-FIB-MC90 [21], which relates to the compressive strength hardening functions:

(8) $$f_{c}\left(t_{e}\right)=k_{c}\left(t_{e}\right)f_{c}\left(t_{s}\right)$$

where *k*_c_ hardening functions, *t_e_* Effective age [days], *t_s_* Standard age, basic age [days], 28, 90, or 365 d, and *f_c_* Compressive strength of cylinders Ø150 mm [MPa],

(9) $$k_{c}\left(t_{e}\right)=exp\left\{s\left[1-{\left(\frac{t_{s}}{t_{e}}\right)}^{0.5}\right]\right\}$$

with s Factor regarding cement type set to 0.38, *t_s_* Basic age 28 d.

The tensile strength of the RCC is related to the compressive strength [22]:

(10) $$F_{t}=0.15f_{c}^{\prime }{}^{0.744}$$

The assessment of the crack occurrence is checked by the calculating the crack index I_cr_ as:

(11) $$I_{cr}=\frac{F_{t}(t)}{\sigma (t)}\geq \;1.0$$

where *σ*(*t*) and *F_t_*(*t*) are the tensile stresses and RCC tensile strength, respectively, at time *t* [6]. The crack index was evaluated at the end of placement time of every RCC lift. So, after the execution of the thermal analysis of the stage under consideration, stress analyses were executed, and the crack index was evaluated using Equation (11). At the end of analysis, the crack index variation over the entire time of analysis can be obtained and the risk of cracking can be clearly recognized.

## 4. Finite Element Modelling

**Figure 3:**
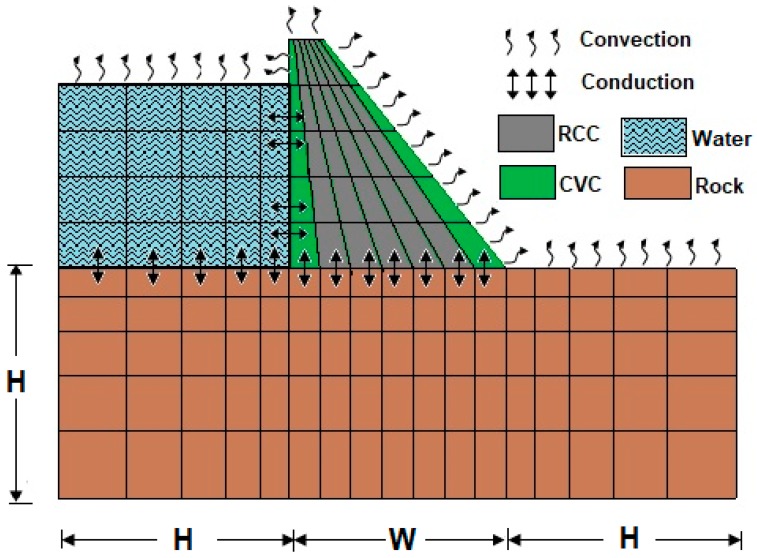
Finite element modeling of Al-Khanaq RCC dam.

The height of Al-Khanaq dam’s highest dam block is 34 m. The 2D finite element mesh model is shown in Figure 3. The eight-node isoparametric element was used in the analysis. The thickness of each RCC layer was taken as 1 m. The upstream and the downstream faces of the dam body are modeled using conventional concrete (CVC). The rock foundation was modeled with a width H (dam height) from both sides (upstream and downstream) and with depth H below the dam base. The finite element method is used to simulate the heat exchange between the impounding water and the upstream side of the dam body where the water finite elements were added in layers similar to the placement of RCC layers according to the reservoir filling schedule. The boundaries at the dam top, upstream, downstream, and reservoir top are exposed to air, so the heat transfer on these boundaries is through convection. When the reservoir filling starts, these boundary conditions will be changed, so there will be heat conduction between the reservoir water and immersed upstream side and the top of the rock foundation. 

### 4.1. Initial Conditions

The temperature distribution in the rock foundation and the RCC placing temperature are the two initial conditions considered in the analysis.

#### 4.1.1. Evaluation of the Foundation Temperature

The temperature distribution of the rock ground was determined using heat transfer analysis. First, we assumed that the initial temperatures of all the nodes of the rock ground are equal to the mean annual air temperature, which is 26 °C in Bisha. Then, the heat transfer was analyzed between the atmospheric temperature and the rock ground for three years [19].

#### 4.1.2. RCC Placing Temperature

The RCC placing temperatures is related to the average annual temperature, current ambient temperature, added processing, aggregate stockpiling, and added mixing energy [23]. Thus, based on the steps summarized in Table 3 in [23] for calculating the RCC placing temperature, Equation 12 is derived and used in this research for calculating the daily RCC placing temperature of Al-Khanaq RCC dam which expressed as:

*RCC_placing_* = 0.11 × *Average_annual_* + 0.22 × *Previous* + 0.67 × *Current* +1.5 (12)

### 4.2. Material Properties

The material properties for the RCC and the rock foundation are adopted from previous studies of the first author [6,7,24] and summarized in Table 1. The cementitious contents of the RCC mix are 100 kg/m^3^ ordinary Portland cement and 100 kg/m^3^ fly ash.

The surface heat transfer coefficient *h* (film coefficient) was applied to all exposed surfaces to represent the convection heat transfer action between the surrounding air and the concrete surface. The surface heat transfer coefficient is calculated as [25]:

(13) $$h=h_{c}+h_{w}$$

where, for a concrete surface, the average value of *h_c_* is 5.7 W/m^2^ ·°C, and *h_w_* is related approximately to the wind speed *v*: *h_w_*= 3.8 *v*, (*v* in m/s). In Bisha, the average monthly wind velocity is 2.0 m/s. Thus, using Equation (13) yields *h* = 13.3 W/m^2^·°C. 

The effect of solar radiation during the construction was incorporated by allowing a 1.0 °C increase in the ambient temperature [26].

### 4.3. Recorded and Estimated Air Temperatures and Wind Velocities

Since the ambient air temperature and the wind velocity are the parameters mainly affecting the thermal response of RCC dams, the recorded temperatures and wind velocities at Bisha airport for a period of five years (2012–2016) were provided by the General Authority of Meteorology and Environmental Protection in Kingdom of Saudi Arabia in terms of the maximum and minimum daily temperature and the daily mean wind velocities.

The hourly air temperatures are estimated from the average maximum and minimum daily temperatures using a sinusoidal curve approximation:

(14) $$T_{air}=\frac{\left(T_{\max}+T_{\min}\right)}{2}+\frac{\left(T_{\max}-T_{\min}\right)}{2}Sin\left(2\pi \left(\frac{\tau -D}{24}\right)\right)$$

where *T*_max_ is the maximum recorded daily air temperature, *T*_min_ is the minimum recorded daily air temperature, τ is the time in hours (1–24), and *D* is the time at which the maximum ambient temperature occurs.

## 5. Results and Discussion 

Using the Finite element modeling of Al-Khanaq RCC dam (Figure 3) and the RCC material properties found in the literature [24], in addition to the Bisha environmental data (temperature and wind velocities), the finite element code developed by Bayagoob [24] for the thermal and structural analysis is used and the obtained results are as follows.

### 5.1. Foundation Temperatures

**Figure 4:**
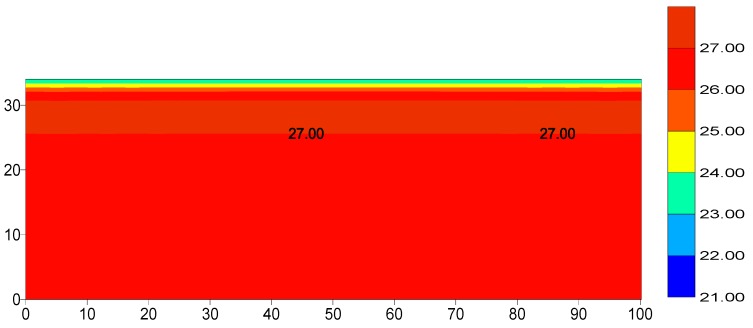
Foundation temperature distribution (°C).

The temperatures of the rock foundation nodes were evaluated using the thermal analysis of the dam foundation for a period of three years using the average maximum and minimum daily air temperature for five years (2012–2016). The temperatures distributions within the ground rock foundation were obtained and are shown in Figure 4. 

Figure 4 shows that the top nine meters of the dam are affected by the convection action of the environment, where the top surface temperature equals the air temperate at the end of analysis, and gradually increased in a downward direction to reach the maximum value of the average annual temperature (27 °C). These foundation temperatures were used as an initial condition in the sequential thermal analysis of the dam body.

### 5.2. Dam Body Temperature

**Figure 5:**
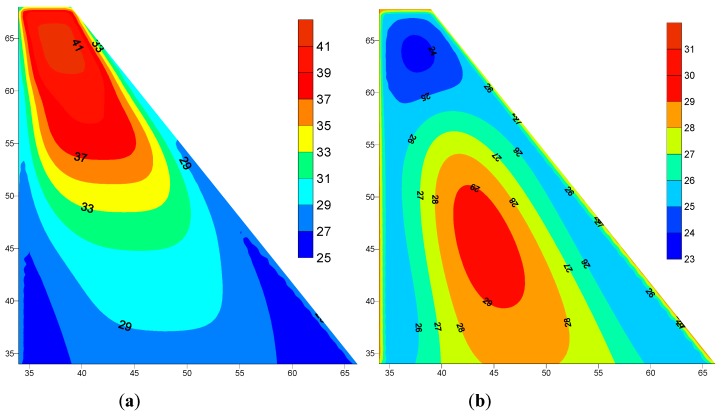
Dam body temperatures distributions (°C): (**a**) immediately after construction and (**b**) after one year of construction.

The dam body node temperatures were evaluated using the Al-Khanaq dam construction plan shown in Figure 2, which started December 1, 2016. The temperature distribution within the dam body immediately after finishing the construction is shown in Figure 5a. The formation of a high-temperature zone occurred at level 63 m, where the construction aligned with the starting of warmer summer days. This zone disappeared after one year of dam construction with an empty reservoir, as shown in Figure 5b, due to the cooling of the dam body with time and the small volume region compared to the large volume at the dam base, where the zones of moderate temperatures still existed at the dam body center as shown in Figure 5b.

**Figure 6:**
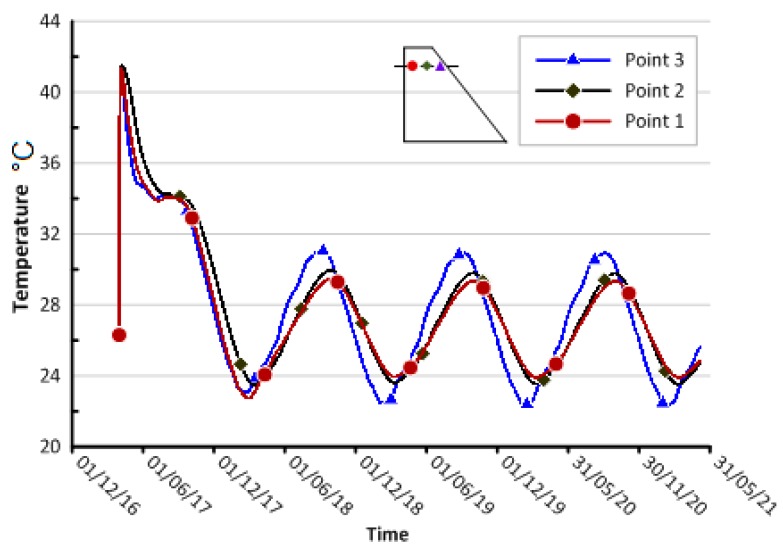
Temperature variation at three middle points at 63 m level.

An attempt was also made to draw the temperature variation with time as shown in Figure 6 for three selected points, equally spaced2.4 m apart, situated within the high temperature zone at the63 m level. From the beginning, all the points traced approximately the same trend of a sinusoidal shape with an initial increasing phase to the peak temperature rise (42 °C). This temperature is approximately equal the sum of the placement temperature (26°C) of the 29^th^ stage and the heat of hydration temperature (18 °C). Then the sinusoidal trends follow approximately the average daily air temperature variation with time. Also, it has been observed in Figure 6 that, there is a slight increase in the maximum and the minimum values of the predicted temperatures of the third point which is located near the downstream side. Moreover, the maximum and minimum values of point three were predicted earlier than those values of point one and two. This could be due to the inclined geometry of the downstream side making the surface area near point three slightly larger than those at points one and two. Thus, point three is more rapidly affected by environmental changes than points one and two.

### 5.3. Rerservoier Temperature:

**Figure 7:**
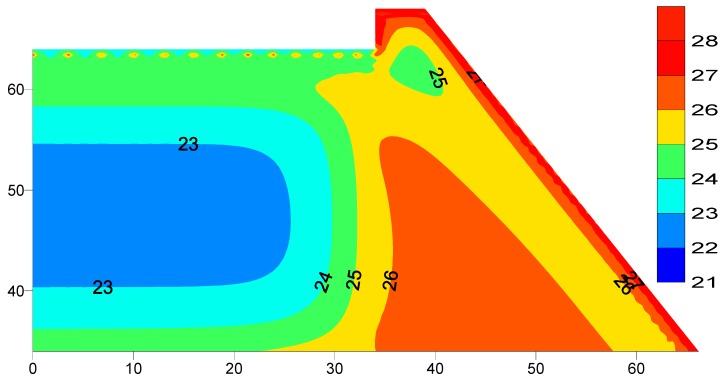
Reservoir dam body temperatures distribution after three years of filling (°C).

The heat exchange between the impounding water and the dam body is simulated using the water interaction modeling described in Section 4. The idealization of the reservoir mesh is set up to the maximum water level of 30 meters using 30 water layers each of one meter thick which equal the thickness of the opposite RCC layers in order to maintain the element continuity. The thermal analysis simulating the reservoir water and dam body heat exchange is performed using a proposed filling period of the dam reservoir of two months and an initial filling temperature of 20 °C. The reservoir filling was proposed to start after one year of finishing the dam construction which corresponds to 13^th^ of April 2017 and ending in 11^th^ of June 2017. The thermal analysis of Al-Khanaq dam was executed a further three years after filling the reservoir. The temperature distribution at the end of the thermal analysis (three years) is shown Figure 7. Inspection of Figure 7 clearly shows that the dissipation of heat from the warm dam body and the rock foundation to the cold reservoir water by conduction action, and the heat gained from the surrounding air through the reservoir surface by convection. 

### 5.4. Stress Analysis

**Figure 8:**
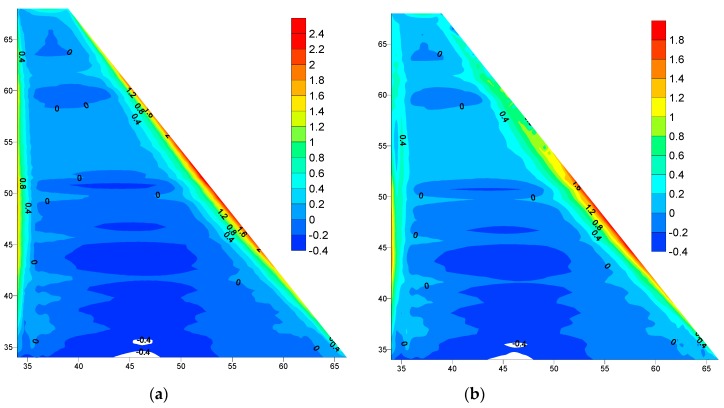
Maximum principle stress distributions with an empty reservoir during first winter December 2017(MPa): (**a**) Normal Placing Temperature, (**b**) Controlled Placing Temperature 20 °C.

Al-Khanaq RCC dam was analyzed after the placing of every RCC layer using the linear elastic material relationship with the plain strain condition for the rock foundation and the plane stress condition for the dam body, considering the variation in the RCC elastic modulus and the tensile strength with time. The analysis was conducted under the effect of thermal loads, dam body self-weight, and hydrostatic pressure. The hydrostatic pressure was applied gradually according to the reservoir filling program which was assumed to take two months starting one year after finishing the dam construction. Thus, the filling rate will be 1 m/2 days for the total 30 m operating level. Figure 8a,b show the comparison between the maximum principle stresses distribution over the dam body predicted during the first winter season (December 2017) after finishing the dam construction with empty reservoir for the normal placing temperature and the controlled 20 °C facing case, respectively.

Figure 8a,b shows the formation of narrow tensile stress zones along the downstream face of the dam. It is clearly observed that the controlling of the placing temperature of the upstream and downstream facing affects the dam stresses where the maximum tensile stress has been reduced from 2.4 MPa to 1.8 MPa.

**Figure 9:**
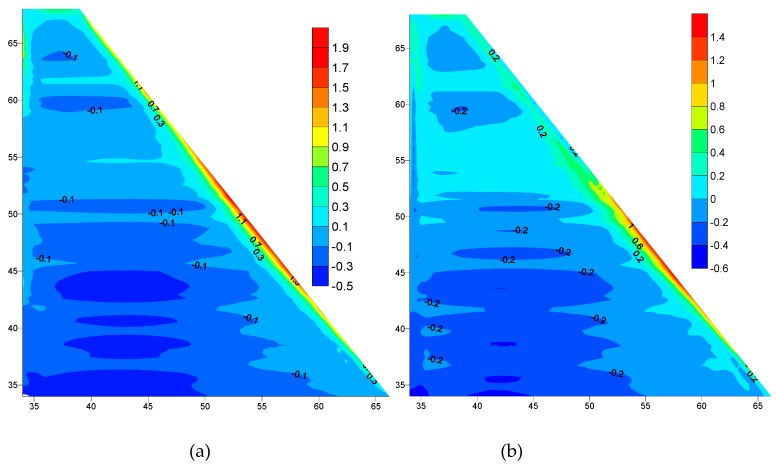
Maximum principle stress distributions with full reservoir during the second winter December 2018 (MPa): (**a**) Normal Placing Temperature, (**b**) Controlled Placing Temperature 20 °C.

Figure 9a,b shows the comparison between the maximum principle stresses distribution over the dam body predicted during the second winter season (December 2018), after filling the dam reservoir for the normal placing temperature and the controlled placing temperature 20 °C case respectively. The maximum tensile stress has been reduced from 1.9 MPa to 1.4 MPa. This is due to the effect of controlling the placing temperature of the upstream and downstream facing. 

In comparison between the empty and full reservoir cases, the tensile stress has been reduced from 2.4 MPa to 1.9 MPa with an empty reservoir and normal placing temperature as shown in Figure 8a and Figure 9a. Also, the tensile stress has been reduced from 1.8 MPa to 1.4 MPa with a full reservoir and controlled placing temperature as shown in Figure 8b and Figure 9b. This reduction is due to the effects of the hydrostatic load, which caused tension on the upstream and compression on downstream of the dam.

**Figure 10:**
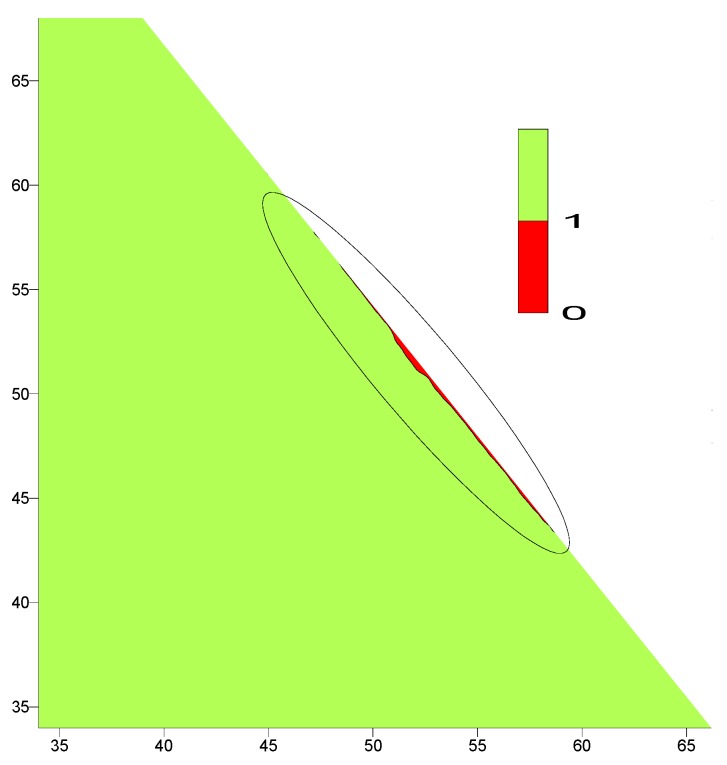
Crack index distribution over the dam body.

To check the dam risk of cracking during and after construction, the crack indices were evaluated at all Gaussian integration points during the whole period of analysis. Figure 10 shows the distribution of the minimum recorded values of the crack indices which indicates that the dam is safe against cracking where their values are greater than 1.0 except for the downstream side where surface cracks have been observed within a small narrow boundary which developed after the construction of the dam and before filling the reservoir during the first winter season.

**Figure 11:**
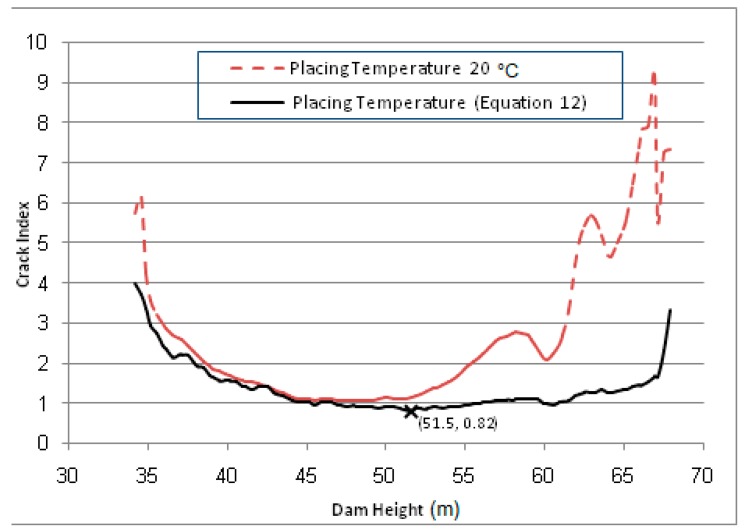
Crack index variation along the downstream outer Gaussian points.

To eliminate the problem of surface cracks developed on the downstream side, the placing temperature of the CVC for the facing of downstream and upstream sides is restricted to 20 °C. Figure 11 shows the comparison (with and without restriction) of the crack index variation along the outermost Gaussian points on the downstream edge for the case of empty reservoir as the worst case observed in Figure 8. It is clear to observe the effect of reducing the placing temperature of the CVC at the downstream and upstream sides on the elimination of the developed surface cracks. The crack index values drops below the limiting value (1.0) during the first winter season in the case of unrestricted placing temperature case compared to the restricted case where the crack index values remains greater than limiting value (1.0). 

**Figure 12:**
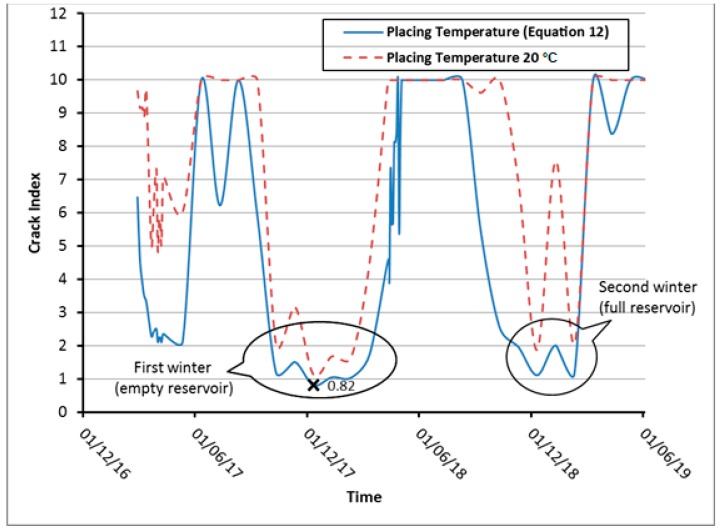
Crack index variation at level 51.5 m and 0.1 m from downstream edge.

Moreover, the crack index variation with time is drawn in Figure 12 for the most observed critical point at which the minimum predicted value of crack index has been observed in Figure 11 (cross marked) and located at level 51.5 m and 0.1 m from the downstream face. It is clearly observed that, the crack occurred in the first winter season where the crack index values drops below the limit value (1.0) compared to the controlled placing temperature case (20 °C) where the crack index values are great than the limit value during and after the dam construction and filling the dam reservoir.

## 6. Conclusions

Based on the results obtained, the following conclusions were drawn: RCC dam technology can be applied safely in arid regions like Bisha state in Saudi Arabia using a suitable starting RCC placement schedule, usually in winter season. Conventional concrete facing must be applied in the upstream and downstream faces of the dam. For construction of RCC dams in Bisha State, the finite element model shows that, the placing temperature of facing conventional concrete of the downstream and upstream sides must be restricted to 20 °C in order to eliminate the risk of thermal surface cracks in the downstream face of the dam. The crack index provides a good indication of the cracking risk of the dam body during and after construction. 

## Figures and Tables

**Table 1:** Thermal and structural material properties.

Material	CVC	RCC	Rock	Water
Heat conduction coefficient, *k*(W/m·°C)	2.8	2.7	2.97	0.615
Heat convection coefficient, *h*(W/m^2^·°C)	13.3	13.3	12	500
Specific heat, *c*(J/kg·°C)	1150	1150	1000	4810
Density (kg/m^3^)	2350	2325	2650	1000
Elasticity modulus, *E*(Mpa)	25000	18,200	24,000	–
Poisson ratio (*ν*)	0.2	0.2	0.2	–

## References

[1] Saetta A., Scotta R., Vitaliani R. (1995). Stress Analysis of Concrete Structures Subjected to Variable Thermal Loads. J. Struct. Eng..

[2] Carvera M., Oliver J., Prato T. (2000). Simulation of Construction of RCC Dams I: Temperature and Aging. J. Struct. Eng..

[3] Carvera M., Oliver J., Prato T. (2000). Simulation of Construction of RCC Dams II: Temperature and Aging. J. Struct. Eng..

[4] Luna R., Wu Y. (2000). Simulation of Temperature and Stress Fields During RCC Dam Construction. J. Constr. Eng. Manag..

[5] Waleed A.M., Jaafar M.S., Noorzaei J., Bayagoob K.H. Effect of placement schedule on the thermal and structural response of RCC dams using finite element analysis. Advanced in geotechnical engineering with emphasis on dams, highway materials, and soil improvement. Proceedings of the Geo Jordan Conference 2004.

[6] Noorzaei J., Bayagoob K.H., Thanoon W.A., Jaafar M. (2006). Thermal and stress analysis of Kinta RCC dam. Eng. Struct..

[7] Jaafar M.S., Bayagoob K.H., Noorzaei J., Thanoon W.A. (2007). Development of Finite Element Computer Code for Thermal Analysis of Roller Compacted Concrete Dams. J. Adv. Eng. Softw..

[8] Gaspar A., Lopez F. (2014). Methodology for a probabilistic analysis of an RCC gravity dam construction. Modelling of temperature, hydration degree and ageing degree fields. Eng. Struct..

[9] Su H., Wen Z., Yan X., Liu H., Yang M. (2018). Early-warning model of deformation safety for roller compacted concrete arch dam considering time-varying characteristics. Compos. Struct..

[10] Zhang X.F., Li S.Y., Chen Y.L., Chai J.R. (2009). The development and verification of relocating mesh method for the computation of temperature field of RCC dam. Adv. Eng. Softw..

[11] Li M., Guo X., Shi J., Zhu Z. (2015). Seepage and stress analysis of anti-seepage structures constructed with different concrete materials in an RCC gravity dam. Water Sci. Eng. J..

[12] Mirzabozorg H., Hariri-Ardebili M.A., Shirkhan M. (2015). Impact of solar radiation on the uncoupled transient thermo-structural response of an arch dam. Sci. Iran..

[13] Cavusli M., Ozolcer I.H., Kartal M.E., Karabuult M., Coskan S. (2017). The effect of reservoir length on the earth quake behavior of roller compacted concrete dams. Online J. Sci. Technol. Jan..

[14] Zhang X., Liu Q., Zhang X., Li Y., Wang X. (2018). A Study on Adiabatic Temperature Rise Test and Temperature Stress Simulation of Rock-Fill Concrete. J. Math. Probl. Eng..

[15] Kuzmanovic V., Savic L., Mladenovic N. (2013). Computation of Thermal-Stresses and Contraction Joint Distance of RCC Dams. J. Therm. Stresses.

[16] Žvanut P., Turk G., Kryžanowski A. (2016). Effects of Changing Surrounding Conditions on the Thermal Analysis of the Moste Concrete Dam. J. Perform. Constr. Facil..

[17] Incropera F.P., Lavine A.S., Bergman T.L., DeWitt D.P. (2002). Fundamentals of Heat and Mass Transfer.

[18] Sergerlind L.J. (1984). Applied Finite Element Analysis.

[19] Ishikawa M. (1991). Thermal Stress Analysis of a Concrete Dam. J. Comput. Struct..

[20] Conrad M., Malkawi A.H. Investigations on the modulus of elasticity of young RCC. Proceedings of the Fourth International Symposium on Roller Compacted Concrete (RCC) Dams.

[21] CEB-FIB (1990). MC90(1990): CEB-FIB Model Code 1990.

[22] Maikawi A.I.H., Mutasher S. A direct tensile strength for roller compacted (RCC) gravity dams. Proceedings of the Fourth International Symposium on Roller Compacted Concrete (RCC) Dams.

[23] Tatro S.B., Bombich A.A., Hess J.R. (2000). Case Histories of Mass Concrete Thermal Studies. Final Report of the Structures Laboratory of the ERDC/SL, TR-00-8, Engineer Research and Development Center (ERDC).

[24] Bayagoob K.H. (2008). Thermal and Structural Analysis of RCC Dams. Ph.D. Thesis.

[25] Duffie J., Beckman W. (1980). Solar Engineering of Thermal Processes.

[26] U.S. Bureau of Reclamation (1997). Thermal Study Mass Gradient Andsurface Gradient Analysis Procedure and Examples, ETL 1110-2-542.

